# Imaging Methods Applicable in the Diagnostics of Alzheimer’s Disease, Considering the Involvement of Insulin Resistance

**DOI:** 10.3390/ijms24043325

**Published:** 2023-02-07

**Authors:** Petra Hnilicova, Ema Kantorova, Stanislav Sutovsky, Milan Grofik, Kamil Zelenak, Egon Kurca, Norbert Zilka, Petra Parvanovova, Martin Kolisek

**Affiliations:** 1 Biomedical Center Martin, Jessenius Faculty of Medicine in Martin, Comenius University in Bratislava, 036 01 Martin, Slovakia; 2 Clinic of Neurology, Jessenius Faculty of Medicine in Martin, Comenius University in Bratislava, 036 01 Martin, Slovakia; 3 1st Department of Neurology, Faculty of Medicine, Comenius University in Bratislava and University Hospital, 813 67 Bratislava, Slovakia; 4 Clinic of Radiology, Jessenius Faculty of Medicine in Martin, Comenius University in Bratislava, 036 01 Martin, Slovakia; 5 Institute of Neuroimmunology, Slovak Academy of Sciences, 845 10 Bratislava, Slovakia; 6 Department of Medical Biochemistry, Jessenius Faculty of Medicine in Martin, Comenius University in Bratislava, 036 01 Martin, Slovakia

**Keywords:** Alzheimer’s disease, insulin resistance, neuroimaging, magnetic resonance volumetry, magnetic resonance spectroscopy, functional magnetic resonance, diffusion magnetic resonance, perfusion magnetic resonance, positron emission tomography, pancreas, liver

## Abstract

Alzheimer’s disease (AD) is an incurable neurodegenerative disease and the most frequently diagnosed type of dementia, characterized by (1) perturbed cerebral perfusion, vasculature, and cortical metabolism; (2) induced proinflammatory processes; and (3) the aggregation of amyloid beta and hyperphosphorylated Tau proteins. Subclinical AD changes are commonly detectable by using radiological and nuclear neuroimaging methods such as magnetic resonance imaging (MRI), computed tomography (CT), positron emission tomography (PET), and single-photon emission computed tomography (SPECT). Furthermore, other valuable modalities exist (in particular, structural volumetric, diffusion, perfusion, functional, and metabolic magnetic resonance methods) that can advance the diagnostic algorithm of AD and our understanding of its pathogenesis. Recently, new insights into AD pathoetiology revealed that deranged insulin homeostasis in the brain may play a role in the onset and progression of the disease. AD-related brain insulin resistance is closely linked to systemic insulin homeostasis disorders caused by pancreas and/or liver dysfunction. Indeed, in recent studies, linkages between the development and onset of AD and the liver and/or pancreas have been established. Aside from standard radiological and nuclear neuroimaging methods and clinically fewer common methods of magnetic resonance, this article also discusses the use of new suggestive non-neuronal imaging modalities to assess AD-associated structural changes in the liver and pancreas. Studying these changes might be of great clinical importance because of their possible involvement in AD pathogenesis during the prodromal phase of the disease.

## 1. Alzheimer’s Disease

Alzheimer’s disease (AD) is one of the most prevalent neurodegenerative diseases and the most common type of dementia. It is characterized by the progressive and nonreversible loss of brain functions, which adversely impacts memory, thinking, language, judgment, and behavior, all of which affect the patient’s personality and social life [1,2]. Generally, AD is the most prevalent form of dementia, possibly accounting for approximately 60% of all cases [3,4]. Symptoms usually develop slowly from mild cognitive impairment (MCI) manifesting as mild forgetfulness and can gradually worsen over the years to moderate, and finally, to severe stage AD accompanied by the inability to perform basic daily life tasks, to recognize family members, to orient in formerly known space, and to understand language [3,5]. However, not every MCI patient develops AD, with a reported yearly conversion rate of 10% to 15% [6]. In 2019, over 55 million individuals worldwide were affected by this debilitating disease, with an increasing tendency and expectancy of up to 82 million cases in 2030 [1,4]. The prevalence of AD is below 1% in people aged 60 years, with an almost exponential increase with increasing age (e.g., 33% in people aged 80 years), making aging the most prominent risk factor for the onset of AD [3]. In the USA, AD has been identified as the fifth leading cause of death in the subpopulation of seniors over 65 years of age [7]. In addition to age, other risk factors related to vascular disease, including hypercholesterolemia, hypertension, atherosclerosis, coronary heart disease, smoking, obesity, and *diabetes mellitus* (DM) have been reported as being associated with AD. The presence of the *APOE ε4* allele has been found to increase the risk of the disease and particularly impacts the age of onset [3,8]. With regard to the genetics of the disease, familial (prevalence below 0.1% [3]) and idiopathic/sporadic forms of AD are recognized. The three genes, namely *APP* (amyloid precursor protein), *PSEN1* (presenilin-1), and *PSEN2* (presenilin-2), are causative to familial AD [8]. Although the cause of a sporadic form of AD remains puzzling, the aggregation of extracellular amyloid-β (Aβ) (resulting in plaques formation) and intracellular neurofibrillary tangles (consisting of hyperphosphorylated Tau protein) in the brain is obviously associated with the disease progression [1,6,9]. Several other pathogenic mechanisms underlie AD manifestation, including neurovascular dysfunction, cell-cycle abnormalities, inflammatory processes, oxidative stress, and lysosomal and mitochondrial dysfunction [3,6]. A new view of AD has emerged based on the finding that the AD brain is in a state of insulin resistance [2,10]. The primary function of insulin is to allow glucose to be processed as a source of energy in the target tissues and organs. In the brain, glucose is not only the major substrate but also an essential signaling molecule that needs to be continuously and permanently supplied to the central nervous system. This supply is ensured by a specific family of membrane proteins known as glucose transporters (GLUTs), namely GLUT1, GLUT2, GLUT3, GLUT4, and GLUT8 [11,12]. The highest impact on glucose uptake is shown by insulin-independent GLUT1 and GLUT3, although insulin-dependent GLUT4 is also important, having a key role in vascular diseases, including DM-related cerebral small vessel diseases [11,12]. Furthermore, a link has been established between insulin resistance, cognitive disorders, and the reduced activity of GLUT4 transporters [13,14]. In several studies, AD has been suggested to be a metabolic disease (insulinopathy), namely DM type-3 [2,15,16]. The current findings strongly indicate that brain insulin resistance leading to long-lasting hyperglycemia causes neural damage and thus increases the risk of developing MCI and AD. Pathological molecular mechanisms leading to AD are complex and difficult to assess in a clinical setting. However, they are often perceived as characteristic structural brain changes traceable throughout the onset and progression of AD by various neuroimaging methods.

## 2. Neuroimaging in the Cognitive Impairment

The key role of neuroimaging methods is to identify specific structural hallmarks of neurological disease and to confirm/exclude the mimicking of or the co-existing forms of other neurological disorders (e.g., stroke, brain tumors) that might subsequently influence treatment efficacy. According to Braak staging [17], the sequential involvement of cortical structures is standardly imaged in AD patients in the following pattern: early involvement of the entorhinal cortex, followed by the limbic system and hippocampus, spreading to the cortex (precuneus in particular) with temporal lobes [5]. However, up to 25% of AD cases can show an atypical clinical presentation, supporting the use of additional advanced diagnostic strategies. [18]. In particular, the distinction of AD from other forms of dementia (e.g., vascular/multi-infarct dementia, frontotemporal dementia, and Lewy body dementia) is clinically challenging [5,19]. Therefore, an exact AD determination is commonly built on the completion of medical examinations, including neuroimaging performed by standard radiological neuroimaging methods such as computed tomography (CT) and magnetic resonance imaging (MRI). However, in order to identify specific symptoms and to rule out other possible pathologies, neuroimaging methods of nuclear medicine are also recommended: mainly positron emission tomography (PET) and, to a lesser extent, single-photon emission computed tomography (SPECT) [5]. Overall, compared with nuclear medicine, radiological methods are usually routinely available, can be performed more cheaply, and do not burden the patient with the radiation dose (here we do not consider the ionizing radiation in the form of X-rays used in CT scanners) originating from the radiologically marked pharmaceuticals that are directly injected into the patient. 

### 2.1. Radiological Neuroimaging

As mentioned above, the characteristic AD pattern involves cortical atrophy, which is usually suitably detected by CT; however, MRI is more sensitive to volumetric changes and can be used to exclude other causes of dementia. Furthermore, because of its noninvasiveness, MRI is concluded to be the most promising neuroimaging modality [20]. The MR phenomenon, as a physical principle, has enabled the development of several MR modalities that are suitable for neuro-assessment and that can be divided into technical subtypes associated with morphology (MRI, MR-volumetry), function (functional MRI (fMRI)), structure (perfusion: arterial spin labeling (ASL), dynamic susceptibility contrast (DSC) MRI, and dynamic contrast-enhanced (DCE) MRI; diffusion: diffusion weighted imaging (DWI) and diffusion tensor imaging (DTI)), and metabolism (MR spectroscopy (MRS)).

#### 2.1.1. CT Neuroimaging

CT takes advantage of X-ray ionizing radiation that passes through variable-dense tissue, is absorbed, and is finally detected by sophisticated computer equipment producing a series of cross-sectional scans of the examined tissue. With regard to excluding the presence of brain tumors, subdural hematoma, or stroke and detecting cerebral atrophy, including the hippocampus region, enlarged ventricles, and cortical sulci, CT is helpful for AD diagnostics [3]. CT has been shown to be capable of revealing an early stage of AD by finding white matter changes that reflect vascular damage in AD [21]. However, structural changes are usually detectable by visual inspection only in the advanced stages of the disease. The most common CT neuroimaging findings associated with AD is widespread cortical atrophy with a thinning of medial temporal lobe structures; however, this AD determination is challenging because of the overlap with normal aging or another form of dementia [3,22].

#### 2.1.2. MR Neuroimaging

MR uses a noninvasive tool comprising a strong magnetic field and radio-frequency pulses that produce detailed soft tissue scans after computer processing. An MRI examination of patients with suspected dementia is recommended as a standard in order to obtain routine MRI brain sequences, including (1) T_1_-weighted MRI (characterized by short echo and repetition times) for the detection of microstructural changes in AD associated with regional Tau burden and local tissue atrophy, (2) DWI to reveal ischemic and hypercellular lesions, (3) susceptibility-weighted MRI (SWI) for hemorrhage detection, and (4) T_2_-weighted (characterized by long echo and repetition times) MRI, especially the fluid-attenuated inversion-recovery (FLAIR) to identify edema, encephalomalacia, and white matter changes [5]. Structural MRI also reveals white matter hyperintensities (with the majority in the frontal lobe), representing demyelination and axonal loss [23,24], and also enables the prediction of mild-to-severe AD, except for the correlation of these hyperintensities with neuropsychological and psychiatric deficits [24]. Furthermore, white matter hyperintensities are frequently considered to be more a sign of vascular dementia, supporting differential diagnosis [23]. Finally, additional MR techniques have been developed to provide useful information about molecular, metabolic, dynamic, or functional changes in AD. 

##### MR-Volumetry

**Figure 1:**
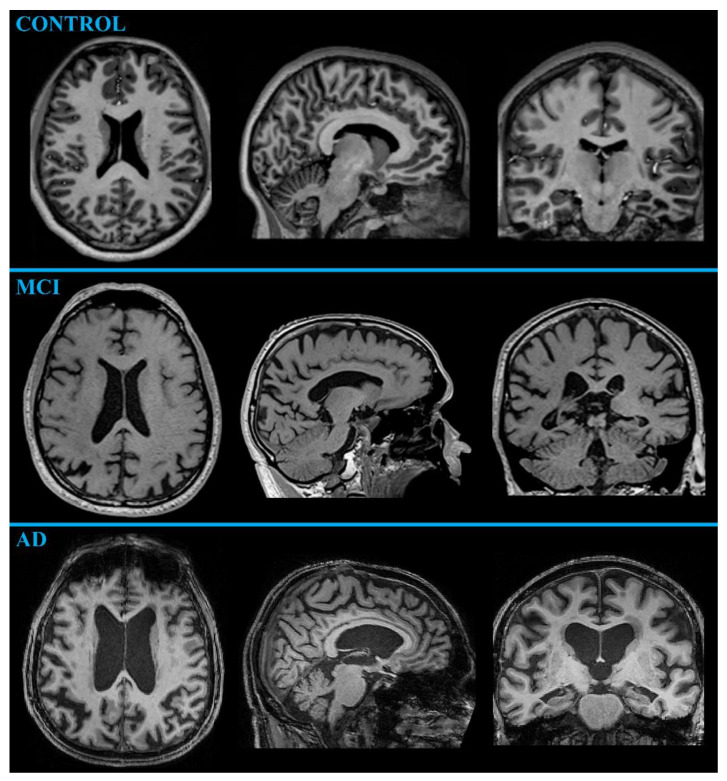
Example of MR neuroimaging (T_1_-weighted MRI at 3 Tesla MR-scanner) of a control subject (70-year-old female), of an MCI patient proceeding to AD in two years (72-year-old male), and of an AD patient (75-year-old female) in axial, sagittal, and coronal sections. The differences are visually subtle, but the increased atrophy in the medial temporal lobe and the enlarged ventricles are apparent in MRI.

MR-volumetry is a suitable method for assessing neurodegenerative deficits because of the wide availability of the relevant equipment, its high evaluation reliability, and the current link between neuronal loss and measurable volume atrophy [19]. An MRI outcome in typical AD is cortical atrophy caused by neuronal loss (Figure 1), usually detectable in the medial temporal (affecting hippocampus, entorhinal, and perirhinal cortex) and parietal lobe (especially the precuneus area), spreading to limbic gray matter structures (including the amygdala, olfactory bulb tract, cingulate gyrus, and thalamus), and finally to the frontal cortical regions [3,5,23]. 

**Figure 2:**
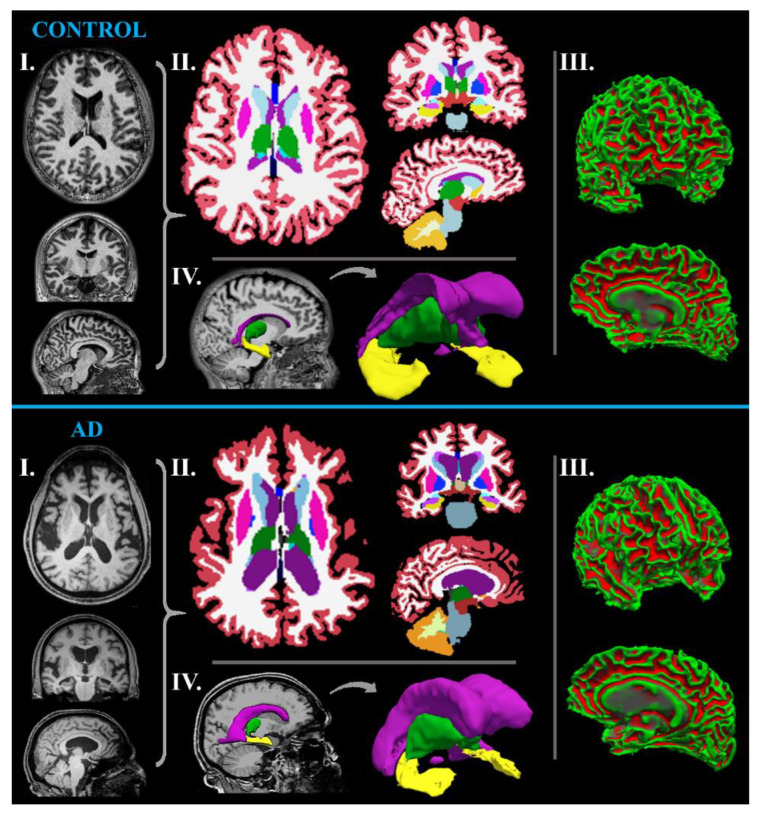
Example of MRI (I.; T_1_-weighted MRI at 3 Tesla MR-scanner) and MR-volumetric (II.; MR-volumetry performed on Freesurfer software) neuroimaging of a control subject (40-year-old female) and AD patient (42-year-old female) in axial, coronal, and sagittal sections. In AD, the apparent atrophy is shown across the white matter (III. 3D model of the whole brain) as well as in several brain structures (IV. 3D model of selected brain areas; atrophy of the hippocampus/yellow, atrophy of the thalamus/green, enlarged lateral ventricles/purple).

The progression of MCI to AD is also reported to be accompanied by greater atrophy in the temporal (especially in the hippocampus) and parietal lobes [23]. However, hippocampal and entorhinal cortex atrophy is also present in other types of dementia, such as frontotemporal and vascular dementia [3]. In a recent study, cortical thickness alone has been reported to allow AD and healthy subjects to be distinguished with an accuracy of 90%, a sensitivity of 96%, and a specificity of 76% [25]. However, brain atrophy is not a specific sign of AD. Nevertheless, several studies have revealed substantial differences in the cerebral atrophy rate in the healthy-aging brain (0.2% per year at the age of 30–50 years; 0.3–0.5% per year at the age of 70–80 years) and in the brain affected by neurodegeneration (2–3% per year) [19]. As further shown by its accelerated volume loss, the hippocampus exhibits a more extensive neuronal loss (~4.5% per year in AD vs. ~1.5% per year in healthy-aging people) among cerebral volumes [26]. Hippocampal atrophy has been reported to differentiate AD patients from normal older adults with 80–90% accuracy [3]. Therefore, hippocampal volume is considered an appropriate marker of AD progression and even seems suitable for monitoring the effectiveness of treatment [25]. Moreover, a tendency has been shown for increased atrophy in the limbic and temporal lobe in AD (Figure 2), whereas normal aging instead affects frontal and parietal gray matter [19]. Even in MCI patients, the atrophy of the medial temporal lobe has been confirmed to correlate with memory decline [25]. In the study of Qian et al. [27], the observed cognitive worsening in MCI patients has been linked to hippocampal atrophy; however, the volume change in the thalamus has been related to non-memory deficits, such as language, executive, and visual-spatial abilities. Thalamic volume atrophy has been observed as an early sign of both MCI and AD, probably preceding the damage to other extra-hippocampal brain regions, such as the amygdala [27]. Furthermore, MR-volumetry might help differentiate AD from dementia with Lewy body and Parkinson’s disease since AD patients manifest more remarkable hippocampal atrophy [23].

##### fMRI

fMRI shows promising potential for detecting alterations in brain function; it benefits from the noninvasiveness of the technique, as it does not require an exogenous contrast agent or radiation exposure [28,29]. It is usually performed by the BOLD (bold oxygen level-dependent) MR method, which evaluates blood flow and volume based on the hemodynamic system that delivers oxygen to active neurons at a greater rate than to inactive neurons. This leads to differences in the ratio of deoxy/oxyhemoglobin, causing a measurable change in magnetic susceptibility [30]. A typical fMRI examination compares MR signals measured during a control situation (e.g., baseline or resting condition) and further in blocks of stimuli (e.g., movement, memorizing, optical stimulation) [16,30]. fMRI studies focusing on episodic memory in AD have demonstrated decreased activation in the hippocampal, parahippocampal, and medial temporal structures during memorizing [23,28]. Although the earliest and most typical clinical symptom of AD is the retention of new episodic memories, fMRI has revealed that many patients with AD also exhibit visuospatial deficits [29]. Moreover, a recent trial examining resting-state fMRI changes in AD has detected worse cognitive improvements in the right gyrus rectus, right precentral gyrus, and left superior temporal gyrus [31]. Research on early and late MCI and task-based fMRI has provided information about the most commonly affected dysfunctions in sensorimotor networks that accompany the worsening of the disease and might be potential biomarkers for the MCI to AD progression [25]. Whereas fMRI has proven disturbances in working memory, visuospatial ability, attention, semantic knowledge, and motor performance in AD, in MCI have been found more dominant changes in attention and working memory [23].

##### Perfusion MRI (ASL, DSC, and DCE) 

Perfusion MR methods yield pharmacokinetic parameters related to tissue perfusion, microvascular vessel wall permeability, and extracellular volume fraction, but neither involves exposure to radioactivity [32,33]. The non-invasive approach in terms of the application of contrast agents is questionable because only ASL employs magnetically labeled arterial blood water molecules as a diffusible flow tracer. Both other methods, DSC and DCE, require the intravenous application of a contrast agent, usually a gadolinium-based contrast agent (Gd-CA). Whereas DSC measures the susceptibility effects of Gd-CA, DCE emphasizes rather the relaxivity effects of Gd-CA on the signal [34]. Anyway, the principle of perfusion MR involves the subtraction of MRI series acquired before and after the perfusion of an endogenous (ASL) or exogenous (DSC, DCE) inherent tracer [33,34]. Differences in blood vessel properties, reduced blood pressure, or increased cerebrovascular blood can be visualized as variable transit times for tracer delivery, resulting in artificial changes in signal intensity [23]. The most commonly used parameter obtained by perfusion MR is cerebral blood flow (CBF; quantified in milliliters of blood per 100 g of tissue per minute), referring to the rate of arterial blood delivery to the capillary bed in brain tissue [32,33]. In particular, AD patients at the same age as controls show a 40% global decrease in CBF [35]. The most prominent decrement in CBF is usually detected in the precuneus, posterior cingulate, and superior parietal cortex, whereas these changes are observed before the tissue atrophy is confirmed [32,33]. Compared with healthy people, typical hypoperfusion for AD patients is found in the posterior cingulate, precuneus, inferior parietal, lateral prefrontal, and temporal cortex, including the parahippocampal gyrus and hippocampus [32]. Similar to AD, hypoperfusion occurs in the occipital, temporal, and parietal lobes in MCI patients. CBF is higher in MCI than in AD, especially in the frontal, orbitofrontal, and hippocampal brain areas, including the thalamus, hippocampus, and amygdala [36]. In addition to the possibility of determining the progression of MCI to AD based on hypoperfusion, some specific patterns between AD and several forms of dementia have been confirmed [33,37]. Compared with AD, frontotemporal dementia demonstrates CBF reduction characteristically in the frontal and insula, whereas, in AD, hypoperfusion is observed in posterior regions, including the precuneus and lateral parietal cortex [33]. Lewy body dementia is associated with a lower degree of hypoperfusion in the temporal areas than AD [37]. Similarly, ischemic vascular dementia causes a more significant increase in CBF than AD, with the most evident differences occurring in the regions with subcortical white matter lesions [38].

##### Diffusion MRI (DWI and DTI)

**Figure 3:**
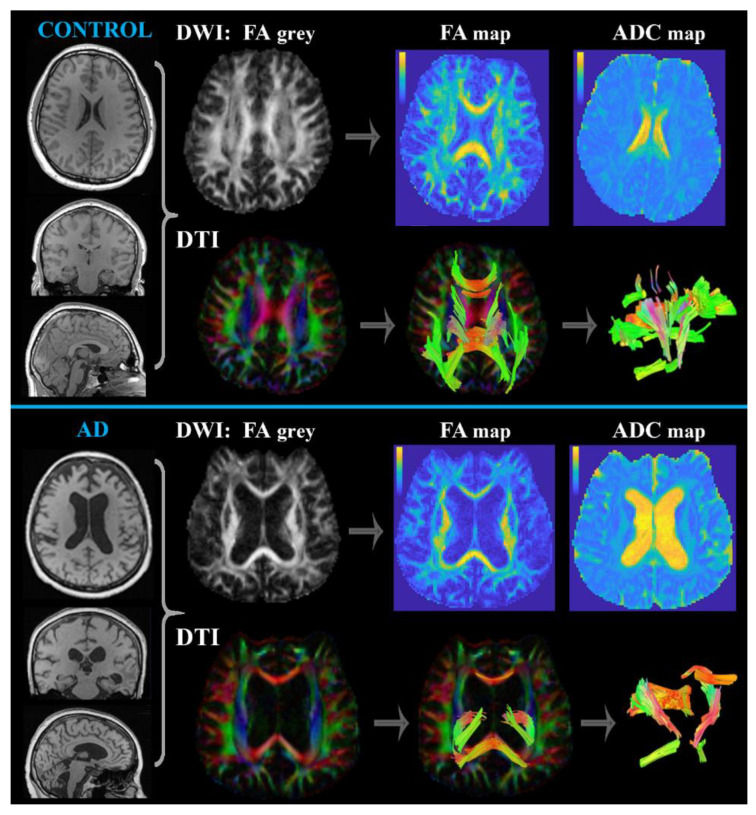
Example of MRI (T_1_-weighted MRI at 3 Tesla MR-scanner), DWI, and DTI (performed on DSI studio software incorporating MATLAB postscripts) neuroimaging of a control subject (50-year-old male) and AD patient (56-year-old female). AD exhibits more restricted diffusion in the tissue, a worse global network density, and lost white matter fiber tracts. Abbreviations: ADC, apparent diffusion coefficient; DTI, diffusion tensor imaging; DWI, diffusion weighted imaging; FA, fractional anisotropy.

Diffusion MRI methods are appropriate to estimate structural neurological integrity defects caused by, for example, neurodegeneration, acute ischemia, tumors, and other lesions, edemas, infections, and protein (Aβ and Tau) plaques [39,40,41]. The methodological principle is based on the measurements of the diffusion of water molecules (random Brownian motion) in the extracellular fluid space. However, water diffusion in the brain is anisotropic because of the presence of axon membranes that limit molecular movement perpendicular to the fibers [19,41,42]. Therefore, the appropriate DWI sequence (with a suitable diffusion-encoding gradient usually expressed by *b*-values 0 and 1000 s/mm^2^ [43]) provides contrast scans based on the measurement of the signal cancellation attributable to diffusion in a given direction [42]. Thus, areas with restricted diffusion motion show less signal loss and become bright in DWI [40,43]. The momentary value of the water molecule diffusion is called the apparent diffusion coefficient (ADC), usually evaluated as two-dimensional ADC maps (Figure 3). In principle, the more restricted the diffusion in the tissue, the smaller the ADC values visualized as hypointensities on the ADC maps [42,43]. The essence of DTI, another related diffusion MRI method, is the graphical representation of the directional diffusion of water molecules along fiber tracts (quantitatively expressed as an FA-fractional anisotropy value varying between 0-maximal isotropic and 1-maximal anisotropic diffusion); provides micro-architectural detail of neuronal connectivity and integrity [41]. FA values can be presented as two-dimensional maps (Figure 3) showing areas with a high degree of anisotropy (high FA value) as bright regions and with dark regions representing low anisotropic diffusion [43]. Studies of the white matter connectivity in patients with various cognitive declines have shown worse global network density, reduced nodal strength, and lower white matter fiber tract integrity associated with poorer memory performance [25,41]. These reflect the changes in synaptoplasticity, which is probably disturbed simultaneously with the progression of the disease. Two hypotheses have been proposed to explain these white matter alterations in AD: (1) Wallerian degeneration upsetting white matter microstructure or (2) diffuse demyelination of the affected tracts [41]. Diffusivity and FA alterations have also been linked to memory deficits and executive dysfunction [23]. Reduced FA and higher tissue diffusivity in AD compared with controls have been reported in the frontal and temporal lobe, posterior cingulate, corpus callosum, superior longitudinal, and uncinate fasciculi [41]. However, a discriminative pattern for AD has suggested lower FA in the parahippocampal cingulum and crus and body of the fornix [44]. Interestingly, higher diffusivity occurs in the parietal and temporal lobes of MCI and AD patients; however, changes in the frontal and occipital regions have been found in AD [23]. Recently, functional brain connectivity, especially in the fronto-limbic circuit, has been suggested to predict the presence of neuropsychiatric symptoms, including AD progression [23,25]. Some significant changes in diffusion metrics are thought to be associated with the presence of the *APOE ε4* allele [41]. Diffusivity alteration might also distinguish AD from other dementias. Lower FA has certainly been found in the frontal areas of patients with frontotemporal dementia, whereas this has not been observed in patients with AD. Nevertheless, higher diffusivity has been detected in parietal and temporal regions in patients with AD compared with those having frontotemporal dementia [23].

##### MRS

MRS is a noninvasive technique involving the MR phenomenon for the in vivo evaluation of concentrations of known biogenic compounds or metabolites from well-defined brain regions without the need for biopsy [20,45,46]. It shares the distinct advantages of the majority of MR methods: it is clinically available, relatively inexpensive, and has no risk of radiation; additionally, MRS has the potential to detect early metabolic changes in brain tissue, whereas structural MRI might not reveal abnormalities. Signals from individual MR-detectable compounds are visualized as spectral peaks resonating at known frequencies shown in parts per million (ppm). Although MRS can be performed based on signals from various nuclei, such as phosphorus-^31^P, carbon-^13^C, and fluorine-^19^F, the most prevalently examined is proton-^1^H because of its natural abundance, higher signal and spatial resolution, and no necessity for additional detection devices [20,47]. In the human brain, ^1^H MRS can detect approximately 25 compounds, among which are those metabolites usually associated with AD [20,45,46]: tCr (creatine-containing compounds), tNAA (N-acetyl-aspartate/aspartyl-glutamate), tCho (choline-containing compounds), mIns (myo-Inositol), Glx (glutamate and glutamine), GABA (γ-aminobutyric acid), and GSH (glutathione).

❖ *tCr (duplet at 3.0 ppm and 3.9 ppm; concentration ~6 mmol/L in the brain [20])*

**Figure 4:**
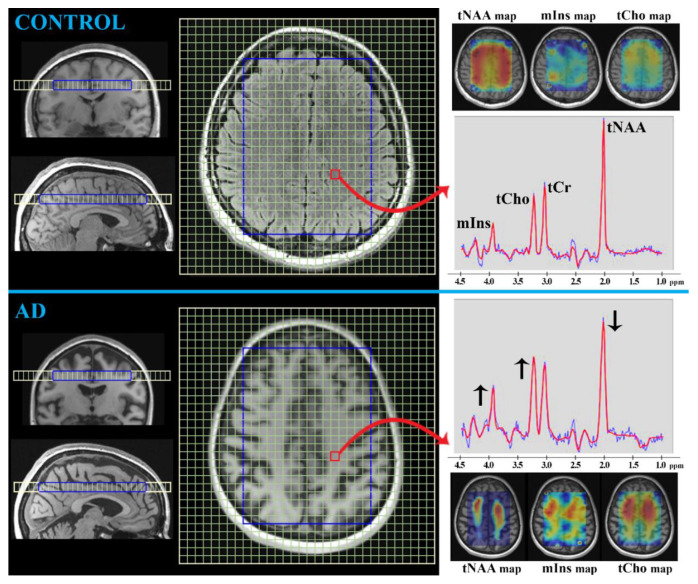
Example of ^1^H MRS neuroimaging (T_1_-weighted MRI at 3 Tesla MR-scanner) obtained by the multivoxel spectroscopy approach with the presented spectra (one selected voxel indicated with the red square and arrow) showing the main metabolite peaks: total creatine (tCr), total choline (tCho), total N-acetyl-aspartate (tNAA), and myo-Inositol (mIns). The ^1^H MRS spectra for a control subject (50-year-old female) and AD patient (55-year-old female) are depicted with the typical metabolic peak changes being indicated by black arrows (↓: decreased, ↑: increased). Metabolic maps of the brain tissue are also shown (tNAA, mIns, and tCho maps).

This peak (Figure 4) represents the signal from creatine and phosphocreatine, indicators of the cellular energetic balance, when considering tCr as an “energetic marker” [20,48]. Since its concentration has been presented as relatively stable across the brain during healthy aging, it is widely used as an internal reference for the relative quantification of other metabolites [46,49]. However, inconsistencies are reported in the tCr levels in several neurological diseases, including dementia [47,48,50]. Nevertheless, the consensus of the majority of authors (except some that are more critical [47,48,50,51]) is that the Cr level remains unchanged across the brain [46,52,53,54,55,56]. Even though the method for relative quantification of metabolite concentration has led to a wide discussion of results, other absolute quantification strategies are technically complicated (e.g., they are affected by the radiofrequency properties of the magnetic coil, by calibration procedures, by spectral fitting methods, by cerebrospinal fluid content correction, by macromolecule suppression, and by spectral editing), all of which might distort the resulting absolute values [46,49].

❖ *tNAA (singlet at 2.0 ppm; concentration ~15 mmol/L in the brain [20])*

Since the signal of tNAA (Figure 4) is attributed to N-acetyl-aspartate and N-acetyl-aspartyl-glutamate (maximally 10% of tNAA), which are predominantly localized in neurons (with a lower occurrence in oligodendrocytes), tNAA is considered as a “neuronal marker”, indicating neuronal viability and density [20,48,54]. Nevertheless, the neurochemical functions of tNAA, especially under pathological conditions, have not yet been clarified; although, it is thought to be involved in the energy metabolism of neuronal mitochondria, in the storage of acetyl coenzyme-A, in the maintenance of neuro-glial signaling pathways and neurotransmission (reservoir for glutamate), and in myelination processes in the brain [20,48]. tNAA can be used as an indicator of neuronal loss/dysfunction, as its levels seem to change in many neurologic and psychiatric disorders [46,47,48]. The usual pattern seen in AD patients is represented by decreased tNAA levels in the parietal and temporal cortex, posterior cingulate, and hippocampus [48,54]. In particular, the hippocampal tNAA/tCr value has been reported to differentiate AD patients from controls [57,58]. Moreover, the declining rate of tNAA is thought to predict cognitive dysfunction [23]. Similarly, a decrease of tNAA/Cr in the medial temporal lobe, primary motor, and sensory cortex has been closely correlated with the Mini-Mental State Examination score [54]. Different tNAA values have been reported in MCI versus AD, although the levels are not always distinguishable from controls, reflecting the different degrees of cognitive deficit [58]. Overall, in AD, compared with MCI, a lower tNAA (or its ratio) has been detected in the hippocampus, temporal, occipital, parietal, and frontoparietal lobes [47,59,60,61]. A reduction in tNAA has even been suggested to predict MCI to AD conversion, reflecting a lower baseline of tNAA in the parietal cortex and further reductions in subsequent measurements [53,62]. The tNAA/tCr value might even be a determinative pattern of AD, considering its higher values in the posterior cingulate gyrus in vascular dementia and Lewy body dementia [47]. A decrease in tNAA/tCr levels has been confirmed in the frontal lobe of frontotemporal dementia and the posterior cingulate gyrus of AD patients [63].

❖ *tCho (singlet at 3.2 ppm; concentration ~2 mmol/L in the brain [20])*

Although the final tCho peak (Figure 4) includes a small amount of free choline and acetylcholine, the main contributors are phosphocholine and glycerophosphocholine, the precursor and breakdown products of membrane phosphatidylcholine [20,48]. Therefore, the clinical significance of tCho has been entrenched as a “marker of membrane turnover”, reflecting the synthesis or degradation of cell membranes [20]. The reports concerning tCho in AD are controversial, showing higher, lower, and unchanged levels, possibly because of a genetic predisposition to membrane turnover (i.e., the presence of the *APOE ε4* allele) [47,64]. Overall, an increased value of tCho or tCho/tNAA is more often reported, especially in the posterior cingulate of AD patients [48,51]. One suggestion for the increasing tCho is that it results from the insufficient acetylcholine production in AD and thus the catabolism of phosphatidylcholine membranes to provide free choline. However, characteristically increased tCho/Cr in the posterior cingulate has been revealed in Lewy body dementia and frontotemporal dementia, compared with controls [54]. Similarly, higher tCho levels have been shown in the frontal and parietal lobes (especially in the white matter) in vascular dementia. In contrast, AD subjects have higher tCho concentrations in the hippocampus and posterior cingulate gyrus [48].

❖ *mIns (multiples at 3.3, 3.5, and 4.0 ppm; concentration ~7 mmol/L in the brain [20])*

The signal of this peak (Figure 4) is composed of free myo-Inositol and myo-Inositol-phosphate and has been designated as a “glial marker” because of their high abundance in glial cells (especially in astrocytes) rather than in neurons [20,48,54]. Several functions are attributed to mIns, such as detoxification, cellular ion regulation, the synthesis of inositol-containing membrane phospholipids, and protein phosphorylation [48,54]. Furthermore, mIns has been established as a crucial growth-promoting factor, a co-factor of several enzymes, a regulator of glucose and osmotic homeostasis, and a messenger in signal transduction [20,65]. Based on a meta-analysis, the usual observation in AD patients is an elevated mIns in the posterior cingulate but not in the hippocampus [48,54]. Increased mIns might reflect gliosis, which is possibly an early AD manifestation that is more pronounced than neural loss or dysfunction (i.e., tNAA decrease). Since both metabolites, namely mIns and tNAA, indicate the most typical changes occurring in AD, tNAA/mIns is understandably considered the best biomarker for AD assessment [55]. MCI patients also exhibit decreased tNAA/mIns in the posterior cingulate gyrus and increased mIns/tCr in the hippocampus [51]. Compared with MCI patients, higher mIns concentrations have been found in AD [23]. Similarly, increased mIns/tCr values have been detected in AD compared with vascular dementia or Lewy body dementia but not frontotemporal dementia [47,54]. A correlation has furthermore been observed between tNAA/mIns and Mini-Mental State Examination score in patients with AD, but no correlation has been seen with vascular dementia subjects [66]. In contrast, AD patients have higher mIns levels in the hippocampus and posterior gyrus than vascular dementia patients, whose increased mIns have been more pronounced in the frontal and parietal cortex [48].

❖ *Glx (multiples at 2.1, 2.4, and 3.7 ppm; concentration ~10 mmol/L in the brain [20])*

**Figure 5:**
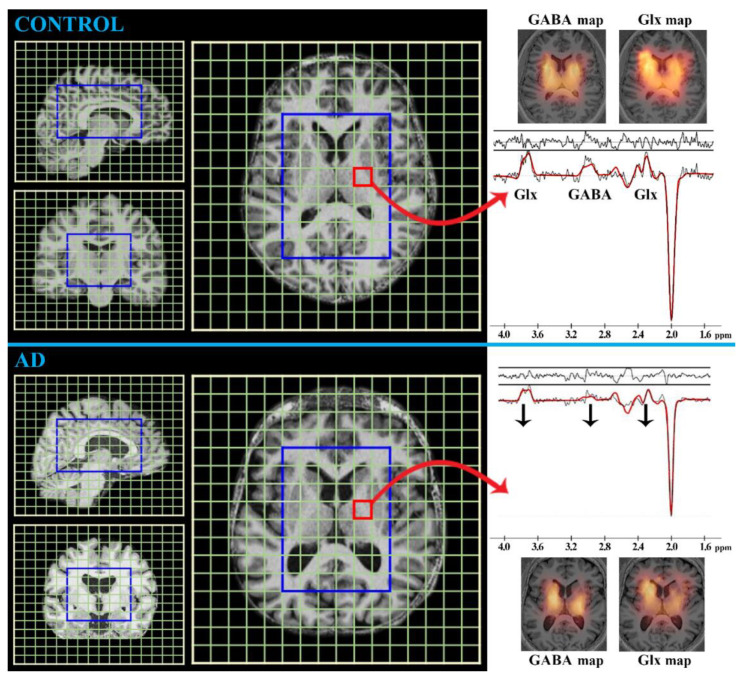
Example of ^1^H MRS neuroimaging (T_1_-weighted MRI at 3 Tesla MR-scanner) measured by the multivoxel Mescher–Garwood-editing MRS approach with the represented spectra (one selected voxel indicated with the red square and arrow) showing the main metabolite peaks: glutamate with glutamine (Glx) and γ-aminobutyric acid (GABA). The ^1^H MRS spectra for a control subject (35-year-old female) and AD patient (42-year-old female) are depicted with the typical metabolic peak changes being indicated by black arrows (↓: decreased, ↑: increased). Metabolic maps of the brain tissue are also shown (GABA and Glx maps).

This peak (Figure 5) consists of hardly distinguishable signals of glutamate and glutamine, which are closely associated with the communication network between neurons, astrocytes, oligodendrocytes, and endothelial and immune cells (i.e., synapse formation, dendrite pruning, cell migration, and differentiation) [20,48]. Even though glutamate cannot cross the blood-brain barrier (BBB), it can be found in all brain cells with the highest abundance in the synaptic vesicles of nerve terminals, from where it can be released into the synaptic cleft [20,67]. Since approximately 85% of cortical neurons are glutamatergic [20,67], glutamate is considered to be the major excitatory neurotransmitter in regulating circadian rhythms and sensory-motoric coordination and controlling emotional, cognitional, and learning functions [20,68]. However, the disturbance in glutamate homeostasis leads to neural hyperexcitability, so-called glutamate excitotoxicity, which evokes a whole range of pathological processes such as the dysfunction of mitochondria and endoplasmic reticulum, an increase in free radicals, microglial activation, and subsequent reactive gliosis ultimately leading to neurodegeneration [20,67,68,69]. Therefore, Glx has been pronounced as a “marker of neuro-excitotoxicity” [20]. The most reported finding in the context of the Glx peak in AD patients is its reduced concentration compared with that in normal brain tissue, especially in the hippocampus, cingulate gyrus, and parieto-occipital white matter [54,70,71]. A lower Glx value has been demonstrated not only in patients with AD but also in healthily aging people (especially in the cingulate gyrus and hippocampus [71]), and in MCI patients [72,73]. Therefore, the detected Glx decrement most likely reflects a decline in metabolic activity in the elderly, leading to cognitive impairment, and might further indicate AD progression. This is further suggested by the finding that decreased Glx/tCr in the hippocampus and cingulate gyrus is more pronounced in AD than in MCI, and both have lower Glx/tCr than in the controls [71]. The same metabolic ratio further seemed to distinguish AD both from vascular dementia with its higher Glx levels and from dementia with Lewy body, which shows a lower extent of affected brain areas [74,75].

❖ *GABA (multiples at 1.9, 2.3, and 3.0 ppm; concentration ~2 mmol/L in the brain [20])*

The primary function of this amino acid in the brain is its role as the major inhibitory neurotransmitter [20,48]. GABA (Figure 5) can be referred to as a “marker of neuro-plasticity” because it plays a unique role in forming action potentials during learning, sensory-motoric processing, and reacting to situations and is therefore essential for psycho-cognitive regulation [20]. Its homeostasis regulates psychological manifestation. On the one hand, normal GABA levels inhibit neuronal hyper-excitability causing irritability, seizures, movement disorders, anxiety, insomnia, fatigue, and various psychiatric disorders; on the other hand, its excessive values cause sedation, sleepiness, and lethargy [76,77,78]. The synthesis of GABA depends on its major precursor, glutamate, forming a vital neurotransmitter cycle, namely the glutamate-glutamine-GABA cycle [20]. However, the detection of GABA is complicated because of interactions between nuclei and signal overlapping, which can be overcome by several methods enabling its quantification, chiefly by Mescher–Garwood-editing of MRS [20,47,71,79]. Several studies have reported decreased GABA in AD, focusing on the temporal, frontal, and parietal lobes [50,77], although no significant GABA changes in AD compared with controls have been confirmed across the frontal lobe, hippocampus, and cingulate gyrus [50,71]. Since the brain regions of AD subjects show changes in Glx but not in GABA, ongoing asymmetric involvement of glutamatergic and GABAergic neurons or different rates of excitation-inhibition might occur in AD-affected cortical areas [80,81]. Furthermore, in assessing GABA changes in AD, we need to consider the age of the patient, because decreased GABA has been confirmed in the cingulate cortex, hippocampus, and frontal and parietal cortex of normal-aging controls [71,77,82]. GABA levels in the frontal cortex have been reported to be correlated with verbal and nonverbal memory and, in the anterior cingulate and occipital cortex, with visuoperceptual functions, supporting the link between the GABAergic and cholinergic neuronal system, a link that is disrupted in AD [77,83]. Since episodic memory decline in individuals is supposed to be a risk factor for AD, GABA might represent an important predictive marker for AD pathology [83]. However, as has previously been mentioned, the relationship between GABA and cognitive perception does not only involve memory. Furthermore, GABA downregulation is not part of a specific pattern for AD; it has also been revealed in the subcortical type of vascular dementia, frontotemporal dementia, or Parkinson’s disease [84,85].

❖ *GSH (multiples at 2.9 and 4.5 ppm; concentration ~1.5 mmol/L in the brain [86])*

The signal of this peak consists of the three amino acids: glutamate, cysteine, and glycine. Although they exist in reduced and oxidized forms, ^1^H MRS applications measure predominantly (with a ratio of 500:1) their reduced conformation [87,88]. Since GSH detection is complicated because of its low abundance and spectral overlapping with other peaks, several signal-editing MRS methods have been developed [86]. In particular, GSH is entitled as a “marker of oxidative stress” because it is the most abundant intracellular antioxidant and free radical scavenger that is responsible for redox balance maintenance and detoxification in oxidative stress caused by reactive oxygen and nitrogen species, free radicals, peroxides, and heavy metals [86,89,90]. Therefore, GLS is thought to be enrolled in oxidative stress–invoked diseases such as neurodegenerative and psychiatric disorders, including AD [90,91]. As expected, in AD, decreased GSH concentrations and increased oxidative stress are correlated with higher Aβ deposition, suggesting GSH as a possible AD biomarker [89]. Reduced GSH levels have been observed in the hippocampus and frontal cortex of AD patients [48,91,92]. Additionally, the decline in GSH levels has been reported to predict the accumulation of Aβ plaques in AD, especially in the temporal and parietal lobes [89,93]. This agrees with several studies presenting GSH as an identifying pattern for AD progression, with the trend of GSH reduction being associated with disease severity [70,90,91]. Related to this is an observation that GSH downregulation accurately discriminates between healthy subjects, MCI, and AD patients [91,92]. These findings suggest early compensatory responses occur in the brain against the increased oxidative stress seen during the early onset of AD [86,89]. Although GSH is not an AD-specific marker, GSH dysregulation has been confirmed in several neurologic disorders, including epilepsy, multiple sclerosis, Parkinson’s disease, and psychiatric disorders [86].

### 2.2. Nuclear Medicine Neuroimaging

PET and SPECT are perfusion imaging methods, with the radiotracers injected into the arteries being delivered to the brain through the BBB. Isotopes, during their decay, emit gamma rays that are collected in detectors and produce 3D scans reflecting brain functions such as regional blood flow, glucose metabolism, abnormal protein deposition, and neurotransmitter deficits [4,6,94,95]. Neuroimaging with SPECT and PET provides valuable information about the functional and molecular pathological processes within the brain many years before the appearance of clinical symptoms of AD; therefore, the results of these methods have been recommended to be considered as diagnostic criteria for the early diagnosis and progression of AD [4,6,95]. Furthermore, both methods are used in differential diagnostics based on their ability to define strokes, seizures, bone illnesses, and infections [94]. Compared with SPECT, PET has advantages such as greater sensitivity (higher detection efficiency of the radioactive emission) and higher spatial resolution (3–4 mm/PET vs. 5–8 mm/SPECT) [18,94]. However, the complex cyclotron production of PET radiotracers and the more sophisticated equipment required by PET makes the cost of PET several times higher than that of SPECT [6,18,94,95]. Furthermore, most positron-emitting radioisotopes in PET radiotracers have shorter half-lives (approximately 6 h/SPECT vs. 2 min/PET radiotracer) compared with the single-photon emitters used for SPECT radiopharmaceuticals, making SPECT radiotracer easily distributable [94,95]. Despite the many advantages of SPECT, this method has been pushed into the background by PET and has become a somewhat experimental method in AD diagnostics, whereas PET has clinically expanded.

#### 2.2.1. PET Neuroimaging

PET uses small amounts of radiopharmaceuticals labeled with positron-emitting isotopes (mostly carbon-^11^C and fluorine-^18^F) to acquire images of functional metabolic radiotracer distribution [6,94,95]. The emitted positron (after traveling at most a few millimeters) collides with an electron from the tissue resulting in annihilation, and the emission of two photons in opposite directions. PET scanners consist of multiple detectors that are located around the patient and that detect the coincidence of this pair of photons. This process makes it possible to infer the position of the emitting of the positrons and to reconstruct tomographic images [18,94]. The most widely used radiopharmaceutical in clinical practice is the glucose analog ^18^F-fluorodeoxyglucose (^18^F-FDG), which allows the direct measurement of regional brain glucose metabolism, and the recently developed radiotracer for Aβ and Tau imaging [3,4,18]. The main problem of protein radiotracers is their off-target binding when they recognize either healthy, undamaged proteins or other misfolded proteins forming beta sheets. However, there is an ongoing effort to improve the selective binding of radiotracers to pathologically changed proteins [96]. Other PET methods based on ^18^F-fluoro-thia-heptadecanoic acid or ^11^C-palmitate tracers are also being tested to research cerebral lipid metabolism [97]. 

##### ^18^F-FDG PET

The positron-emitting ^18^F substituting a hydroxyl group on the glucose molecule is taken up by glucose transporters, phosphorylated, and accumulated in metabolically active cells [5]. In general, glucose metabolism responsible for cerebral activity depends on brain tissue perfusion, which might be affected before any degeneration occurs and thus might enable early AD diagnosis and its differentiation from other types of dementia [6]. A typical early-onset AD pattern on ^18^F-FGD topography is that of hypometabolism in the parietotemporal region, precuneus, and posterior cingulate cortex [3,18]. Hypometabolism involves first the frontal cortex, then increases with the progression of the disease, and usually expands to the sensorimotor cortex and occipital region, as generally corresponds to the atrophic changes depicted on structural images [5,18]. A differentiating sign of MCI to AD progression has been suggested to be hypometabolism in the posterior parietal cortex, precuneus, and posterior cingulate [3,6]. In addition, regional hypometabolism in ^18^F-FDG is considered to be a better predictor for MCI to AD progression than results from MR-volumetry [3]. Moreover, typical patterns of some forms of dementia can be distinguished based on ^18^F-FDG. For example, vascular dementia shows a well-defined decrease in ^18^F-FDG metabolism in a specific vascular territory, usually in the subcortical gray matter structures. In contrast, AD has more typical hypometabolism in the posterior temporoparietal cortex [98]. Similarly, frontotemporal dementia, in which hypometabolism involves the frontal and temporal lobes but spares the occipital lobe and precuneus, can be differentiated from AD, in which both the latter regions are typically affected [5]. Lewy body dementia is characterized by decreased ^18^F-FDG metabolism in the occipital lobe without the involvement of the posterior cingulate gyrus, which is almost invariably hypometabolic in AD [5,99].

##### PET with Aβ Radiotracer

Since Aβ accumulation is generally accepted as being the characteristic pattern of AD, various radiotracers have been developed, such as the thioflavin-T analog called ^11^C-labeled Pittsburg compound B (^11^C-PiB) and the fluorinated analogs called ^18^F-florbetaben, ^18^F-florbetapir, and ^18^F-flutemetamol, all of which enable the imaging and quantification of Aβ depositions [3,18,95]. 

***^11^C-PiB*** does not bind Lewy bodies or neurofibrillary tangles, thus enabling selective quantification of cerebral amyloidosis [4,100]. Therefore, a typical AD pattern has been established for ^11^C-PiB uptake, with the highest retention in the frontotemporal cortex and cingulate regions having histologically confirmed larger amounts of Aβ plaques [3,4,5]. Currently, the majority (50–80%) of MCI subjects who are shown to be Aβ-positive by PET have been found to progress to AD; however, the Aβ-negative PET results represent a low risk of AD progression (0–10%) [6,18]. Unfortunately, the Aβ radiotracer for PET is not without its limitation; a persisting problem is the high prevalence of false-positive cases in older subjects (with a rate of 10% at the age of 60–70 years and 50% at the age of 80–90 years), a complication for establishing causality between the presence of Aβ deposits and AD deterioration in elderly patients [101]. Nonetheless, PET is beneficial in diagnosing AD as opposed to frontotemporal dementia with its lack of ^11^C-PiB uptake compared with high radiotracer uptake in early- and late-onset AD [6].

**Figure 6:**
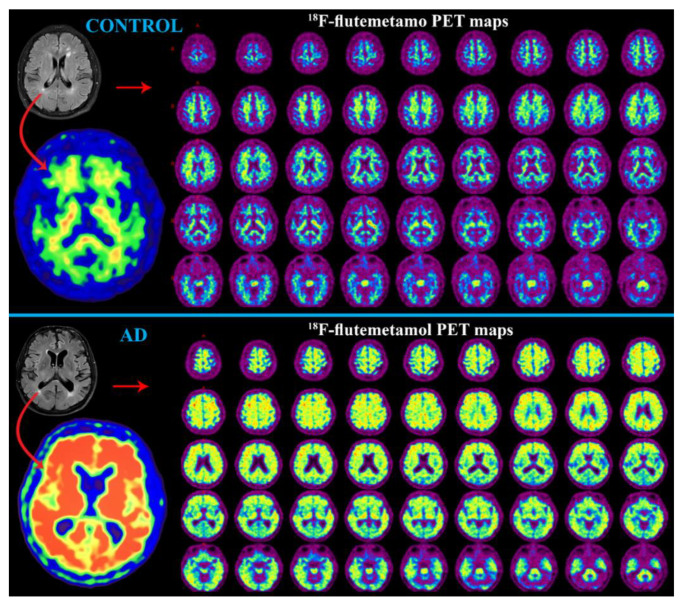
Example of ^18^F-flutemetamol (Vizamyl) PET neuroimaging of a control subject (73-year-old male) and AD patient (72-year-old male). Note the visualization of subtle cortical atrophy with enlarged ventricles (T_1_-weighted MRI at 3 Tesla MR-scanner) and increased retention of the Vizamyl radiotracer demonstrating the presence of Aβ deposition in the AD brain tissue, compared with that of the control subject.

***^18^F-labeled*** amyloid radiotracers, ^18^F-florbetapir and ^18^F-florbetaben are both stilbene derivatives, whereas another radiotracer, ^18^F-flutemetamol (Figure 6), is derived from ^11^C-PiB [4,94]. Overall, the ^18^F-labeled amyloid radiotracers show similar features to ^11^C-PiB but have the great benefit of a longer half-life [4,95]. These radiotracers have been widely used in AD assessment, showing increased radiotracer uptake in cortical areas, including the frontal, temporal, occipital, parietal, cingulate, and precuneus regions, in AD patients than in healthy individuals [102,103,104]. Like ^11^C-PiB, the ^18^F-florbetapir Aβ-positivity in MCI patients is correlated with AD progression [105]. Whereas ^18^F-florbetapir has been reported as a suitable radiotracer for distinguishing cognitively normal young and elderly controls and individuals with AD, another radiotracer, ^18^F-florbetaben, has been proposed as being appropriate for differentiating AD from frontotemporal, vascular, and Lewy body dementia [106].

##### PET with Tau Radiotracer

Neurofibrillary tangles are the pathologic hallmark of neurodegenerative disorders known as tauopathies, among which AD represents the most significant cause of morbidity and mortality [4,5]. Nuclear medicine neuroimaging of neurofibrillary tangles is technically challenging since six isoforms of abnormal Tau protein exist with different ultrastructural conformations and multiple posttranslational modifications [107]. The radiolabeled radiotracer naphthol (^18^F-FDDNP) has been widely used in AD, although it has a nonspecific binding affinity to Tau and to Aβ protein [6,108]. Moreover, several quinoline-derived radiotracers have been developed (e.g., ^18^F-THK523, ^18^F-THK5105, ^18^F-THK5117, ^18^F-THK5317, ^18^F-AV1451) with a higher affinity for Tau than for Aβ [4,6,107]. In general, higher Tau-radiotracer than that in healthy controls has been observed in the orbitofrontal, lateral parietal, and temporal cortices, and in the posterior cingulate and hippocampus of AD patients, correlating with their cognitive impairment [4,6]. Since the presence of pathological Tau protein aggregates in the AD-affected brain is associated with disease severity, the neuroimaging of neurofibrillary tangles should also reflect AD progression and cognitive decline. Indeed, greater Tau radiotracer retention has been shown in AD with early onset compared with late onset [109]. Furthermore, based on Tau uptake, imaging has also made it possible to determine the severity of disease progression with the radiotracer uptake for MCI patients lying between that for AD patients and normal controls [4,6]. In addition, differences in Tau retention have been reported to be linked with the presence of the *APOE ε4* allele. The uptake of ^18^F-AV1451 radiotracer is lower in the parietal and occipital cortex and higher in the entorhinal cortex of AD patients with a positive rather than a negative presence of the *APOE ε4* allele [110].

#### 2.2.2. SPECT Neuroimaging

SPECT, compared with PET, involves the use of other types of detectors because of the different types of radioisotopes present in the administrated radiotracers. In principle, SPECT radioisotopes decay straightforwardly giving one gamma ray, that passes through the collimator and forms a pixel in the detector during reconstruction [111]. In SPECT, applied gamma-emitting radioisotopes are typically iodine-^123^I, metastable technetium-^99m^Tc, xenon-^133^Xe, thallium-^201^Tl, and indium-^111^In [94]. The most extensively used radiotracers are ^99m^Tc-ECD (technetium-99m-ethyl cysteinate diethylester) and ^99m^Tc-HMPAO (technetium-99m-hexamethyl propylene amine oxime) [6,18]. Neuroimaging based on these blood flow radiotracers depends on cortical perfusion and thus can reveal hypometabolic regions as a corresponding decrease in tissue perfusion [18,94]. Other promising SPECT approaches have been developed for Aβ neuroimaging [6,94].

##### SPECT Perfusion

In particular, hypoperfusion in the posterior cingulate, posterior parietal cortex, and precuneus has been documented during the early onset of AD, with a spreading tendency into the posterior temporoparietal and frontal cortices providing the high discriminative pattern for MCI to AD progression [6,18]. Anterior temporal and frontal hypoperfusion is a typical deficiency in patients with frontotemporal dementia, as opposed to the posterior temporoparietal SPECT pattern determining AD [18,98]. However, various posterior cortical alterations have been reported in frontotemporal dementia and atypical AD frontal involvement, making it challenging to discriminate between the two clinical entities [112]. Lewy body dementia also shows a similar SPECT finding to that for AD and is represented by hypoperfusion in the posterior parietotemporal cortex. However, the typical extension of Lewy body dementia to the occipital cortex, with the highest involvement in the primary visual cortex is distinguishable. The visual cortex is not affected in AD, which is considered to be the SPECT criterion for the differential diagnosis between Lewy body dementia and AD [18].

##### SPECT with Aβ Radiotracer

An ongoing research undertaking is to develop SPECT agents for imaging Aβ plaque [95,98]. Several gamma-emitting radioisotopes have been shown to be suitable for Aβ plaque binding; however, the technical challenge is their poor ability to cross the BBB [6,94]. ^123^I-based radiotracers such as ^123^I-ioflupane, ^123^I-IMPY, and ^123^I-ABC577 seem to be promising [5,6,113,114], with the last-mentioned showing a high binding affinity for Aβ in a similar context to that of PET Aβ radiotracers [114]. Furthermore, this radiotracer determines AD patients based on its retention in the frontal cortex, temporal cortex, and posterior cingulate compared with normal subjects [6,94]. Another SPECT radiotracer, ^123^I-ioflupane, exhibits decreased uptake in the putamen and spares the caudate head in Lewy body dementia, in contrast to the situation in AD, in which it shows normal striatal uptake [5].

### 2.3. Advantages, Disadvantages, and New trends in Neuroimaging of AD

Neuroimaging is a critical component in the secondary prevention, diagnosis, and treatment of Alzheimer’s disease. Among known methods, CT and MRI are still considered to be the most powerful in neurological practice with respect to revealing detailed morphological information about the brain tissue. Both structural radiological methods are able to reveal typical AD widespread cortical atrophy and thinning of medial temporal lobe structures. However, in favor of MRI are its non-invasiveness, higher sensitivity, and lack of radiation risk (further critical advantages and disadvantages of neuroimaging methods are listed in Table 1).

Other over-standard MR methods (Table 1) sharing benefits of MRI might be used to uncover some typical AD patterns. These are: MR volumetry (evaluating exact cranial atrophy), fMRI (revealing decreased activation of cognitive-affecting brain areas), MRS (detecting metabolic alternations such as decreased tNAA connected with neuronal dysfunction, increased mIns reflecting reactive astrogliosis, elevated tCho caused by neuronal membrane catabolism, altered Glx and GABA linked to disbalanced neuronal activity, and decreased GSH due to ongoing oxidative stress), diffusion MRI (showing worsening in the global network density as reduced FA and higher tissue diffusivity, lower/hypointense ADC and DWI, and lower white matter fiber tract on DTI), and perfusion MR (proving tissue hypoperfusion and reduced CBF).

Despite some disadvantages of nuclear medicine neuroimaging methods (Table 1), PET and SPECT are increasingly being used for definitive AD assessment. Both radiotracer-based perfusion methods are successfully used to assess the presence and/or quantitative aspects of the molecular hallmarks of AD (Tau protein and Aβ) in vivo. The overall more popular PET enables the identification of defects in cerebral activity based on glucose hypometabolism and the detection of amyloid plaques and neurofibrillary tangle depletion by higher Aβ- or Tau-radiotracer uptake. SPECT, the less preferred method, can also detect hypometabolic cortical regions due to radiotracer hypoperfusion or Aβ aggregation neuroimaging based on Aβ-radiotracer uptake.

Each neuroimaging method contributes to understanding the pathophysiology of AD manifestation and is therefore the preferred choice in clinical practice and preclinical research. Finding a reliable predictor of neurophysiological worsening (i.e., clinical behavioral observations or self-reported observations) has become critical in neuropsychiatry. A promising tool seems to be the identification and classification of AD using multi-modality neuroimaging data and an artificial intelligence approach [115,116,117]. Chen et al. [115] provided an excellent overview of various types of machine learning and multimodal data fusion, as well as conceptual and practical challenges and opportunities for future psychiatric disease research. The deep learning method has been sufficiently applied directly in AD using (1) resting-state electroencephalography [116], (2) a convolutional network of multi-modality brain imaging (i.e., MRI, ^18^F-FDG PET, ^18^F-florbetapir PET) [117], or by using (3) automated MRI-based software tools to assess entorhinal cortex thickness, hippocampal volume, and supramarginal gyrus thickness [118], all of which have high sensitivity and specificity for differentiating MCI from AD. Based on the multi-modal artificial machine learning algorithm, several non-unambiguous (i.e., non-specific, non-predictive, or non-distinguishable) neurological examinations (Table 2) have the potential to become meaningful.

## 3. AD as Insulinopathy

**Figure 7:**
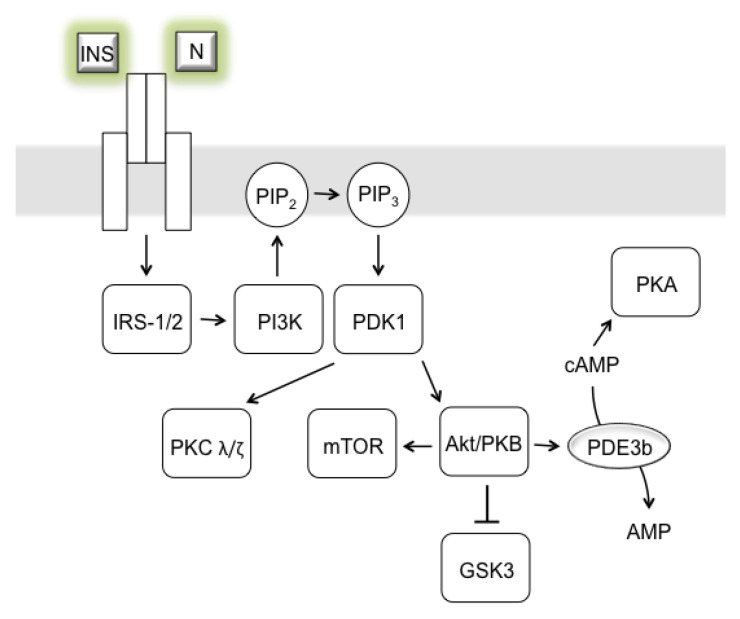
Insulin signaling pathway. Transmembrane structure depicts insulin receptor complex sensing insulin (INS) and neuritin (N; a member of the neurotrophic factor family, promotes neuritogenesis, neuronal survival, and synaptic maturation). Abbreviations: Akt/PKB, Akt protein kinase B; AMP, adenosine monophosphate; cAMP, cyclic adenosine monophosphate; GSK3, glycogen synthase kinase-3 β; IRS-1/2, insulin receptor substrate 1 and 2; mTOR, mammalian target of rapamycin; PDE3b, phosphodiesterase 3B; PDK1, phosphoinositide-dependent protein kinase 1; PI3K, phosphoinositide 3-kinase; PIP_2_, phosphatidylinositol 4,5-bisphosphate; PIP_3_, phosphatidylinositol (3,4,5)-trisphosphate; PKA, protein kinase A; PKC λ/ζ, protein kinase C λ/ζ.

Tau protein accumulation and the Aβ plaque hypotheses are the most frequently quoted to address the pathology behind AD. However, recently an exciting hypothesis suggesting that regional (brain) insulin resistance triggers the onset and progression of AD has been proposed [14]. Insulin is an essential anabolic hormone that maintains peripheral glucose homeostasis [11,14]. Although the brain has, for decades, been considered an insulin-insensitive organ, insulin is now clearly seen to play an essential role in a plethora of brain physiological processes (e.g., regulation of glucose homeostasis; energy metabolism; neuronal differentiation, maturation, and proliferation) and in the regulation of psychic processes (e.g., anxiety, depression) and cognitive functions (e.g., execution, attention, learning, memory) [11,16,76]. Insulin is known to be a prominent activator of the protein kinase B (Akt/PKB) signaling node (Figure 7), which is a principal regulator of pro-proliferative, pro-growth, and anti-apoptotic pathways essential for tissue regeneration, including the remodeling of dendrites and synapses [14,123,124]. Insulin-dependent mitochondrial biogenesis is regulated by phosphatidylinositol-3-kinase (PI3K) – Akt/PKB signaling through the inhibition of glycogen synthase kinase-3β (GSK-3β) [125]. Therefore, the inhibition of GSK-3β increases mitochondrial dynamics, attenuates mitochondrial permeability, and promotes mitochondria-dependent apoptosis [126]. Conversely, GSK-3β hyperactivity is a causal factor in progressive neurodegenerative and psychiatric conditions. It has been shown that overexpression of GSK-3β leads to cognitive impairment and AD [127,128,129].

Despite the importance of insulin, the brain was originally assumed to be largely independent of insulin from the peripheral circulation [11]. However, the transport of insulin from peripheral circulation into the brain is now known to be secured by a highly regulated, saturable transport mechanism (receptor-mediated endocytosis or receptor-mediated transcytosis) that occurs in the vascular endothelium and allows insulin to cross the BBB [2,11,76]. Its derangement has been found in ailments such as obesity, starvation, hyperglycemia, DM, and AD [130,131]. Insulin is also centrally synthesized directly in the brain, in subpopulations of cortical and hippocampal neurons and neural progenitor cells [14,16,132]. The expression of insulin inversely correlates with AD progression according to Braak’s scale [133]. Several studies utilizing ^18^F-FDG PET have also confirmed that insulin resistance is related to lower glucose metabolism, which is further linked to declining memory functions and even connected to AD [97]. Liu et al. [134] have demonstrated that, in rat hippocampal neurons, neurotoxic Aβ-derived diffusible ligands induce abnormal insulin receptor expression and insulin signaling, which consequently might contribute to the central insulin resistance seen in AD [134,135]. Aβ itself can be degraded by a variety of peptidases, including insulin degrading enzyme (IDE), which represents the further relation between hyperinsulinemia, insulin resistance, and AD [14,136]. Recently, Lauer et al. [137] demonstrated a link between Aβ-production and Aβ-degradation forming a regulatory cycle in which APP intracellular domain (AICD) promotes Aβ-degradation via IDE and IDE itself limits its own production by degrading AICD. When brain tissue passes into a state of insulin resistance, hyperglycemia arises, a condition characteristic of DM type-2 [2]. Moreover, several other pathophysiological conditions have been reported to be associated with insulin resistance, such as oxidative stress, insulin signaling disorder, mitochondrial dysfunction, neuroinflammation, accumulation of glycosylation-end products (i.e., glycated proteins or peptides), and metabolic syndrome [2,14,15]. The link between AD and DM type-2 has been suggested by a simple correlation showing that a significant proportion of AD patients suffer from DM type-2 or systemic insulin resistance [2,10,138]. Specifically, the onset of DM type-2 before the age of 60 doubles the risk of AD, and every 5 years of living with DM type-2 increases the risk of the development of AD by 24% [139]. DM type-2 has been further concluded to correlate with reduced brain perfusion and BBB fitness, disrupting the transfer of substances between the periphery and the brain [140]. Similarly, an early BBB dysfunction has been shown before AD-related neurodegeneration and cognitive impairment occurs [140,141,142]. BBB breakdown induces neurodegenerative pathophysiological processes such as chronic inflammation, oxidative stress, deterioration of mitochondrial homeostasis, and related damage to the energy metabolism of neurons, sentencing them to gradual death [141,143]. Although neuronal loss can be compensated to a large extent at the level of synaptoplasticity [20], excess loss over a critical limit can manifest itself as the onset of clinical AD symptoms and their progression [140,143]. In connection with the function of BBB and the etiopathology of AD, insulin has been found to protect pericytes from the toxic effect of Aβ [143,144]. Pericytes are cell types that occur in capillaries and are of fundamental importance in regulating various microvascular functions such as angiogenesis, BBB fitness, capillary blood flow, and the transfer of immune cells into the brain [145]. They also form part of the glial scar, isolating damaged parts of the brain, and have stem-cell-like properties [146]. Only recently, pericytes have been shown to play a crucial role in the pathogenesis of many neurological disorders, including DM type-2 and AD [145,147]. Another high-risk factor for AD development is obesity, which often precedes the onset of DM type-2 [138]. Obesity further contributes to vascular damage and BBB breakdown [140,148]. Current evidence suggests that a single insulin dose can reverse the increase in brain-soluble Aβ and in behavioral impairment induced by a long-term high-fat diet [148,149]. Adiponectin, an adipokine produced primarily by adipose tissue cells, but also in muscles, regulates fatty acid metabolism and glucose levels and has recently been found in the brain [150]. Increased adiponectin expression has been shown to correlate with a reduced risk of developing DM type-2 [151,152]. On the other hand, long-term adiponectin deficiency leads to a loss of cell sensitivity to insulin, a loss caused by the inactivation of adenosine monophosphate-activated protein kinase (AMPK) [153]. AMPK is an essential component of insulin signaling; in rodent muscles, it phosphorylates and activates the insulin receptor, suggesting a direct link between AMPK and the insulin signaling pathway [154]. Although the phosphorylation of the insulin receptor by AMPK in human neurons is disputed, this kinase is directly involved in the pathophysiological processes leading to the hyperphosphorylation and aggregation of Tau protein [155].

### 3.1. Suggestive non-Neuronal Imaging in AD

If AD is an insulinopathy, neuroimaging might uncover only a part of the clinical image. The study of patients suffering from DM, dyslipidemia, or insulin resistance with early signs of MCI might reveal important biochemical and structural hallmarks in organs strongly correlated with systemic insulinopathy, namely the pancreas and liver. The appearance of these hallmarks might well precede the onset of AD and, thus, be crucial for the development of AD preventive measures and treatment.

The liver is an integral part of the body’s metabolism. It has a prominent and direct impact on the functional performance of the brain, primarily via the regulation of pyruvate levels required for glucose production and the maintenance of brain energy homeostasis [14,156]. The latter has been elegantly demonstrated by Serres et al. [157]. They showed that pyruvate recycling (observed through the labeling of brain metabolites) mainly originates from peripheral (liver) metabolism [157]. Furthermore, the liver modulates the concentration of glutamate, a prominent neuromodulator (responsible for neuronal synaptoplasticity) and neurotransmitter [14,158]. The potential involvement of peripheral (liver)/central glutamate homeostasis in the development of neurodegenerative disorders has been recently reviewed by Oanolapo et al. [159]. It was also demonstrated that the liver adipose tissue secretes several proinflammatory factors, such as interleukin 6 (IL-6) and tumor necrosis factor alpha (TNF-α), which play a role in inducing systemic insulin resistance. In AD patients, IL-6 is implicated in the formation of APP. Both IL-6 and APP contribute to neuroinflammation and neurodegeneration [14,160].

The pool of available data points toward the strong linkage between the development and onset of AD and liver dysfunction and the consequent systemic metabolism pathologies [161,162]. Nho et al. [163] have correlated aberrant liver function (represented by the activities of serum-based liver enzymes alanine aminotransferase (ALT) and aspartate aminotransferase (AST)) in 1,500 AD patients to more significant brain atrophy detected by MRI, disrupted brain glucose metabolism measured by ^18^F-FDG PET, and increased Aβ accumulation showed by ^18^F-florbetapir PET. Their study further suggests that the monitoring of the liver status (especially via plasma AST/ALT ratio) is indicative of patients with a higher risk of AD. Other studies have shown a strong association between insulin resistance and hepatic steatosis (affecting up to 70% of patients with DM type-2), which biochemically presents as altered ALT and AST values [164,165]. Therefore, the pathological accumulation of lipids in hepatocytes (liver steatosis), the infiltration of inflammatory immune cells in the liver parenchyma, and the secretion of pro-inflammatory cytokines resulting in liver damage might be involved in the development of AD [161,162].

DM does not occur without substantial impairment of intrapancreatic insulin secretory capacity [166]. Recent reports acknowledge that not only an impairment of pancreatic functions but also pronounced morphological changes (tissue atrophy) are hallmarks of DM type-1 and DM type-2. Pancreas atrophy has been postulated to occur in response to the loss of the trophic effect of hyperglycemia or to be attributable to the chronic inflammation associated with progressive beta cell destruction [167]. Further, increased levels of fatty acids have been demonstrated to cause pancreatic beta cell de-differentiation, a mechanism underlying the development and onset of DM type-2 [166,168]. Thus, the exact loss of the pancreatic volume and the fat content in the pancreas should be quantified, not only in strictly diabetic patients but prospectively also in prediabetic patients. In agreement with the latter proposal, pancreatic islets of diabetic patients have been shown to accumulate deposits of islet amyloid polypeptide that are structurally similar to Aβ precursor proteins in the brain of AD subjects [169,170]. Janson et al. [169] showed that islet amyloid is more frequent and extensive in AD patients than in non-AD controls, and that diffuse and neuritic plaques are not more common in DM type-2 patients than in control subjects. Moreover, they demonstrated that if diffuse and neuritic plaques are present in DM type-2 patients, duration of DM type-2 correlates with their density [169]. Therefore, AD has recently been speculated to predispose patients to DM type-2, which is in accordance with the observation that diabetic patients suffer more frequently from AD when compared with the non-diabetic population [169,171]. Additional multicentric (global) studies should be conducted to provide clear conclusions.

#### 3.1.1. Liver Imaging Linked with AD

For decades, the standard method for determining the fatty liver score was hepatic needle biopsy, a method that suffers from the disadvantage of being invasive. Currently, imaging techniques such as transabdominal ultrasound (US), CT, and a wide range of MR methods have become more common for in vivo liver assessment [172]. Although US is the first-line imaging test for liver assessment because of its cost-effectiveness, safety, and availability, its specificity and sensitivity are highly variable. The measurement itself is influenced by operator experience and confounded by the surrounding body fat in the case of obese patients [173,174,175]. The greatest disadvantage of CT hepatic examination is its ionizing radiation that limits regular screening of liver diseases [172,175]. MR has a clear advantage over both US and CT based on its non-ionizing features, higher sensitivity of the detection of liver fat, lower variability, and higher reproducibility [172,175,176]. The common hepatic MRI examination consists of T_1_- and T_2_-weighted MRI for assessing hepatic lipid and/or iron content but can be extended by gadolinium-enhanced T_1_-weighted MRI for grading more severe acute hepatitis and fibrosis and for delineating vascular abnormalities [177,178]. Another increasingly applied method is MR elastography (MRE), the principle of which is based on an image-encoding response of the stimulated soft tissue produced by harmonic mechanical vibrations and the reconstruction of parameters denoting viscoelastic (i.e., the degree of liver stiffness) tissue properties [174,175,179]. Hepatic MRE enables an examination of the entire liver and is suitable as a screening method for liver fibrosis [177,179,180]. The variety of MR modalities facilitates the evaluation of abnormal liver fatty infiltration (e.g., T_1_-weighted fast spoiled gradient echo [178], MRI-estimated proton density fat fraction [180], in-phase and opposed-phase imaging [174], and Dixon sequences or ^1^H MRS [181]) based on distinguishing the fat–water composition within soft tissues. A prognostic marker for hepatic steatosis seems to be MR-estimated proton density fat fraction, defined as the ratio of protons bound to fat and water [181]. In this concept, ^1^H MRS has been considered to be more accurate than MRI methods and shows a close correlation with histological results [174,181]. The liver ^1^H MRS spectrum is represented by a water singlet peak resonating at 4.7 ppm effectively quantifying lipid (triglyceride) peaks echoing as multiples at 0.9-1.1 ppm (methyl -CH_3_ protons) and 1.3–1.6 ppm (methylene -CH_2_ protons) [174,182]. Globally, the increasing lipid peaks relative to the water peak correlate with increasing steatosis grade [174,182]. In addition to liver lipids choline-containing compounds (tCho singlet at 3.2 ppm) can be visualized, in ^1^H MRS spectra, representing metabolite alterations in focal hepatic malignancies [174]. Increased tCho in liver tissue is suggested to be associated with elevated cell membrane turnover, cell proliferation, and carcinogenesis [183]. The ratio of tCho/lipids is an especially useful marker for differentiating benign and malignant liver tumors representing the proportion of cell proliferation (biosynthesis of membrane phospholipids) to cell necrosis (membrane breakdown) [183]. Finally, many modified MRI-based techniques, including diffusion and perfusion MRI or magnetization transfer, have been developed for staging liver fibrosis [177].

#### 3.1.2. Pancreas Imaging Linked with AD

Visualization of the pancreas by standard US is difficult because of its small size, irregular shape, and challenging position, all of which are usually aggravated by overlying bowel gas or surrounding fat, especially in obese patients [184,185]. However, these issues compensate for CT imaging being handicapped by ionizing radiation unsafe for repeated or screening use [186]. Overall, a variety of MR technologies can effectively execute pancreatic imaging; however, difficulties are encountered inherent to MR field susceptibility and motion artifacts [185,186,187]. Quantitative MRI techniques, mainly T_1_-weighted MRI and DWI, based on differentiating tissue fat and water signals in the pancreas, have become prognostic clinical tools for characterizing and staging pancreatic diseases, including acute and chronic pancreatitis steatosis, or pancreatic cancer [186,188,189,190]. However, to date, MRI of the pancreas has a limited spatial resolution that does not enable the image of the very small pancreatic islets, which are directly involved in DM pathogenesis [185]. Nevertheless, MRI can delineate the borders of the pancreas and can thus quantify its volume, which is, in both types of DM, lower than in healthy subjects [184]. In addition, several MR methods enable the establishment of pancreatic fat composition, as protons associated with water and of the primary methylene groups of triglyceride fatty acid chains with slightly different frequencies [187]. Recent research suggests that ^1^H MRS and fat–water MRI methods (i.e., Dixon, IDEAL) are especially suitable for rapid, robust, and accurate characterization of the fat and water content in entire organs [186]. Quickly resolved peaks of water, tCho, and lipids are seen in pancreatic ^1^H MRS spectra, enabling lipids to be distinguished in adipocytes and in triglyceride droplets and cytosol [187,191]. Generally, the higher the pancreatic fat concentration, the more the reduced beta cell function and the risk of DM type-2 development [185]. MR perfusion methods also seem to be essential tools, enabling the characterization of the dense pancreatic network of capillaries with a high rate of blood flow that is altered in dysfunctional islet vasculature as a typical sign of DM [185].

## 4. Conclusions

Neuroimaging (in particular, the basic methods of radiology and nuclear medicine) remains a fundamental diagnostic step toward identifying AD and monitoring its progression. Furthermore, new technological modalities of neuroimaging combined with modern artificial intelligence and machine learning approaches have further potential to advance the diagnostic and prognostic algorithms in identifying neurodegenerative disorders and monitoring their progression or efficacy of treatment regimens.

Recent advances in the understanding of AD pathoetiology indicate that AD is not purely a neurodegenerative disease but rather a highly complex metabolic disease affecting not only the central nervous system but also other organs such as the liver and pancreas. Therefore, pathological changes at the level of their functions and structures might be of great importance, mainly because of their involvement in AD pathogenesis during the prodromal phase of the disease. Furthermore, disturbed insulin homeostasis and glucose metabolism associated with MCI and AD led to the hypothesis that AD could be an insulinopathy. This has triggered an outburst of research, bridging the molecular pathophysiology of the pancreas, liver, and brain in MCI and AD, which has resulted in the identification of prominent overlaps between, at first sight, very distant disease units, namely AD and DM. This new way of looking at AD pathoetiology also makes a reevaluation of the diagnostics, treatment, and preventive strategies for AD an urgent undertaking.

In light of the foregoing, it becomes obvious that the use of comprehensive examinations combining above-standard neuroimaging methods with suggestive non-neuronal imaging, of at least the liver and pancreas, may strengthen the diagnostic algorithm in MCI patients, early AD patients, and patients at a higher risk of cognitive impairment due to DM and prediabetes. Moreover, further identification of relevant combinations of radiological and biochemical/molecular markers is much needed to ensure the quality and widespread accessibility of standardized AD diagnostics and monitoring of disease progression.

## Figures and Tables

**Table 1:** An overview of the most distinctive advantages and disadvantages of neuroimaging methods applicable for AD assessment. Abbreviations: ASL, arterial spin labeling; CT, computed tomography; DCE, dynamic contrast-enhanced magnetic resonance imaging (MRI); DSC, dynamic susceptibility contrast-enhanced MRI; DTI, diffusion tensor imaging; DWI, diffusion weighted imaging; EMA, European Medicines Agency; EU, European Union; fMRI, functional MRI; MRS, magnetic resonance spectroscopy; PET, positron emission tomography; SPECT, single-photon emission computed tomography.

Methods	Principal Advantages	Principal Disadvantages
CT	short examination time; less critical contraindication criteria	X-ray exposure; contrast agents may worsen kidney function; poorer definition of critical brain structures (hippocampus, brain stem)
MR methods	no radiation exposure; less expensive examination; widespread availability; wide range of examinations; (standardly) no contrast agents	relatively poor pathological specificity; contraindication criteria (presence of magnetic non-compatible medical devices or foreign bodies); uncomfortable with noise and limited size of MR-gantry
MRI	high-resolution morphology	insensitive to small amount of calcification and bone fracture
MR-volumetry	sensitive tissue volume changes	several non-standardized softwares
fMRI	disrupted brain functions	challenging tasks for attention
MRS	non-invasive detection of metabolic changes	robustness; difficult absolute quantification; non-unambiguous predictive or distinguishable biomarkers
diffusion MRI (DWI, DTI)	demyelinated axons; damaged nerve tracks; 3D visualization of neural pathways	pitfall of mucinous or hemorrhagic lesions; highly motion sensitive
perfusion MRI (ASL, DCE, DSC)	acute inflammation; tissue degradation; ! in the case of ASL no contrast agent	susceptibility artifacts; ! in the case of non-ASL methods (DCE, DSC) contrast agent
PET and SPECT	definitive diagnostics; functional and molecular pathological processes	radiation exposure; more expensive examination; not routine equipment; Tau tracer (Tauvid^©^) not registered by EMA and thus not commercially available in EU

**Table 2:** The overview of non-common neurological examinations applicable for AD assessment. Abbreviations: ECoG, electrocorticography; EEG, electroencephalography; MEG, magnetoencephalography; SWI, susceptibility-weighted magnetic resonance imaging; TSC, transcranial sonography.

Methods	Utility	Principal Advantages	Principal Disadvantages
EEG [115,116]	neuronal electrical activity is measured via electrodes positioned on the scalp	low cost examination; easy and non-invasive performance	multiple signal distortions
ECoG [115]	intracranial EEG that measures signals directly from the cortical surface	excellent spatial and spectral resolution minimizing inter-electrode spacing	invasive performance; necessity of surgery
MEG [115,116]	EEG measured as magnetic fields produced by brain and evaluating by superconducting quantum interference devices	non-invasiveness; high temporal and spatial resolution	technically chalenging (necessity of specialized shielding to eliminate the magnetic interference)
TSC [119]	evaluating brain tissue echogenicity through an intact skull bone using ultrasound technique	non-invasiveness; low cost examination	insufficient temporal acoustic bone windows; diagnostic precision depends on neurosonographers skills
T_2_-relaxometry [20,120]	T_2_ relaxation time (one of MRI measurable parameters) is sensitive to microstructural changes (i.e., Aβ, Tau, and iron deposition; changes in water homeostasis)	non-invasiveness; early changes detection also in deep brain structures	sensitivity to magnetic susceptibilities, magnetic field variation, and inhomogeneities
SWI [121,122]	MRI method suitable for the detection of microhemorrhages that may indicate cerebral amyloid angiopathy	non-invasiveness; easy (routine) and fast examination of the whole brain	difficult to differentiate small venous structures from small hemorrhages and thrombosis

## Data Availability

Not applicable.
